# Assessing the feasibility, acceptability, and fidelity of a tele-retinopathy-based intervention to encourage greater attendance to diabetic retinopathy screening in immigrants living with diabetes from China and African-Caribbean countries in Ottawa, Canada: a protocol

**DOI:** 10.1186/s40814-023-01372-5

**Published:** 2023-09-09

**Authors:** Valerie Umaefulam, Mackenzie Wilson, Marie Carole Boucher, Michael H. Brent, Maman Joyce Dogba, Olivia Drescher, Jeremy M. Grimshaw, Noah M. Ivers, John G. Lawrenson, Fabiana Lorencatto, David Maberley, Nicola McCleary, Sheena McHugh, Olivera Sutakovic, Kednapa Thavorn, Holly O. Witteman, Catherine Yu, Hao Cheng, Wei Han, Yu Hong, Balkissa Idrissa, Tina Leech, Joffré Malette, Isabelle Mongeon, Zawadi Mugisho, Marlyse Mbakop Nguebou, Sara Pabla, Siffan Rahman, Azaratou Samandoulougou, Hasina Visram, Richard You, Junqiang Zhao, Justin Presseau

**Affiliations:** 1 Clinical Epidemiology Program, Ottawa Hospital Research Institute, Ottawa, Canada; 2 Department of Ophthalmology, Maisonneuve-Rosemont Ophthalmology University Center, Université de Montréal, Montreal, QC Canada; 3 Department of Ophthalmology and Vision Sciences, Donald K Johnson Eye Institute, University Health Network, University of Toronto, Toronto, Canada; 4 Department of Family and Emergency Medicine, Université Laval, Québec City, Canada; 5 VITAM, Centre for Research On Sustainable Health, Université Laval, Québec City, QC Canada; 6 Department of Medicine, University of Ottawa, Ottawa, Canada; 7 Women’s College Research Institute, Women’s College Hospital, Toronto, Canada; 8 Department of Family and Community Medicine, University of Toronto, Toronto, Canada; 9 School of Health & Psychological Sciences, City, University of London, London, UK; 10 Centre for Behaviour Change, University College London, London, UK; 11 Department of Ophthalmology, The Ottawa Hospital, Ottawa, Canada; 12 School of Epidemiology and Public Health, University of Ottawa, Ottawa, Canada; 13 School of Public Health, University College Cork, Cork, Ireland; 14 Division of Endocrinology & Metabolism, St. Michael’s Hospital, Faculty of Medicine, University of Toronto, Toronto, Canada; 15 Dalla Lana School of Public Health, University of Toronto, Toronto, Canada; 16 Patient Local Advisory Group, Ottawa, Canada; 17 Centretown Community Health Centre, Ottawa, Canada; 18 West Ottawa Specialty Care, Ottawa, Canada; 19 School of Nursing, University of Ottawa, Ottawa, Canada; 20 School of Psychology, University of Ottawa, Ottawa, Canada

**Keywords:** Diabetic retinopathy, Screening, Tele-retinopathy, Immigrants, Protocol, Fidelity, Acceptability, Feasibility, Theoretical domains framework, Theoretical framework of acceptability

## Abstract

**Background:**

Diabetic retinopathy is a leading cause of preventable blindness in Canada. Clinical guidelines recommend annual diabetic retinopathy screening for people living with diabetes to reduce the risk and progression of vision loss. However, many Canadians with diabetes do not attend screening. Screening rates are even lower in immigrants to Canada including people from China, Africa, and the Caribbean, and these groups are also at higher risk of developing diabetes complications. We aim to assess the feasibility, acceptability, and fidelity of a co-developed, linguistically and culturally tailored tele-retinopathy screening intervention for Mandarin-speaking immigrants from China and French-speaking immigrants from African-Caribbean countries living with diabetes in Ottawa, Canada, and identify how many from each population group attend screening during the pilot period.

**Methods:**

We will work with our health system and patient partners to conduct a 6-month feasibility pilot of a tele-retinopathy screening intervention in a Community Health Centre in Ottawa. We anticipate recruiting 50–150 patients and 5–10 health care providers involved in delivering the intervention for the pilot. Acceptability will be assessed via a Theoretical Framework of Acceptability-informed survey with patients and health care providers. To assess feasibility, we will use a Theoretical Domains Framework-informed interview guide and to assess fidelity, and we will use a survey informed by the National Institutes of Health framework from the perspective of health care providers. We will also collect patient demographics (i.e., age, gender, ethnicity, health insurance status, and immigration information), screening outcomes (i.e., patients with retinopathy identified, patients requiring specialist care), patient costs, and other intervention-related variables such as preferred language. Survey data will be descriptively analyzed and qualitative data will undergo content analysis.

**Discussion:**

This feasibility pilot study will capture how many people living with diabetes from each group attend the diabetic retinopathy screening, costs, and implementation processes for the tele-retinopathy screening intervention. The study will indicate the practicability and suitability of the intervention in increasing screening attendance in the target population groups. The study results will inform a patient-randomized trial, provide evidence to conduct an economic evaluation of the intervention, and optimize the community-based intervention.

**Supplementary Information:**

The online version contains supplementary material available at 10.1186/s40814-023-01372-5.

## Background

Diabetic retinopathy is a leading cause of preventable blindness among adults in Canada [1]. Clinical practice guidelines recommend annual diabetic retinopathy screening for people with diabetes to reduce the risk and progression of vision loss [2]. Screening involves dilated ophthalmoscopy and retinal imaging by an optometrist, an ophthalmologist, a retina specialist, or other trained health practitioners. Screening facilitates early retinopathy detection and management and is one of the most effective and least costly ways to reduce the progression of severe eye complications associated with diabetes [3]. Diabetic retinopathy screening and treatment, such as laser treatment, eye injections, and surgeries to prevent sight-threatening complications, are of economic benefit [4–7]. However, retinopathy screening attendance rates are low in Canada. For instance, a cohort study across 5 provinces showed that 38% of people with diabetes have never had a retinopathy examination, and 30% had not had an examination in the last 2 years [8]. Only half of individuals with newly diagnosed type 2 diabetes between 1996 and 2007, in Ontario, Canada, received retinopathy screening within the first year [9]. Data from 2019 shows that of the 1,346,578 adults over 20 years of age with diabetes in Ontario, 34% had not had their eyes screened in the last 2 years [10].

The Canadian 2016 census data show that 21.9% of the Canadian population are foreign-born, and recent newcomers to Canada (individuals who immigrated within the past 5 years) represent 3.5% of the total population [11]. Most of these newcomers (61.8%) were born in Asia, with 10.6% coming from China [12]. Diabetic retinopathy is associated with non-white ethnicity [13], and people from China and Africa have a higher risk of developing diabetes-related complications relative to people of European descent [14]. Screening rates are lower in minority groups, and immigrants who are members of these communities are less likely to be screened than non-immigrants in Canada [14], including people arriving from China, Africa, and the Caribbean. For example, a study in Ontario showed that new immigrants had an approximately 25% lower odds of attending retinopathy screening [9]. In Canada, diabetic retinopathy accounts for 25% of vision loss in people of visible minorities, compared to 4% across all ethnicities in Canada [1].

There is a clear need to better support immigrants to Canada from cultural and linguistic minority groups to attend diabetic retinopathy screening. This can be done by utilizing approaches that bring retinopathy screening to the community and foster greater access, though access alone is not the only barrier. Indeed, numerous factors hinder diabetic retinopathy screening uptake in Canada. For instance, wait times are a challenge to access ophthalmology services in Canada, sometimes taking months [15]. Although optometrists can also conduct retinopathy screening, which costs are/are not covered by optometrist screening are not always clear, sometimes leading patients to pay for retina imaging without advanced notice. Our research with multiple cultural and linguistic minority groups in Canada shows that this lack of cost transparency is a barrier to screening [16]. Also, there is a lack of clarity about the difference between attending eye tests for vision correction (glasses/contacts) and screening for retinopathy.

Tele-retinopathy screening is an alternative, task-shifted approach, whereby the eye screening itself is conducted by a trained eye screening technician rather than an optometrist or ophthalmologist, while the grading (i.e., interpretation of retinal images and diagnosis where indicated) is conducted remotely and asynchronously by an ophthalmologist who can initiate follow-up and treatment where needed. Advances in lower-cost, portable imaging equipment enable the capture of digital retina photographs and optical coherence tomography (OCT) images, which can be uploaded to a secure server for remote interpretation by an ophthalmologist to make management recommendations (such as repeat imaging at a prescribed interval, urgent referral for treatment of sight-threatening diabetic retinopathy). Community-based screening sessions also provide opportunities for further integration of eye screening into broader diabetes education programs. Tele-retinopathy screening programs can detect non-proliferative diabetic retinopathy with > 95% sensitivity and specificity and increase accessibility for patients in urban and rural settings [17, 18]. Additionally, tele-retinal screening achieved a high accuracy for the detection of referable diabetic retinopathy with a specificity of about 95% and a sensitivity of 85% [19]. There are also cost benefits; compared to traditional exams, tele-retinopathy screening is associated with fewer health system costs and greater screening rates in the general population [20]. For example, a cost analysis of a Toronto-based tele-retinopathy screening program showed that the cost per case of retinopathy correctly detected was $379 for tele-retinopathy screening, compared to $985 for standard screening [21].

Many barriers identified in our work with immigrants to Canada from multiple cultural and linguistic minority groups [16] can be addressed by tele-retinopathy screening, for instance, concerns about cost (all testing provided free to patients), access (convenient locations and availability at times outside usual office hours for optometrists and ophthalmologists), and wait times (for specialist ophthalmology services). Nonetheless, our research highlights a range of additional barriers beyond access, time, and cost [16, 22] (Table 1).

**Table 1:** Summary of barriers to diabetic retinopathy screening

Resource	Barriers
**Systematic review with the general population** [23]	Environmental context and resources: access, competing priorities, financial concerns, specialist availability, scheduling appointment, referral issues
	Social influences: doctor-patient communications, language, trust, stigma, community/family support
	Knowledge: awareness of diabetes-retinopathy link, confusion between retinopathy screening and routine eye exam
	Memory/attention/decision processes: symptoms, co-morbidities, forgetting
	Beliefs about consequences: worry about harmful effects of screening, perceived necessity of screening
	Emotions: fear, defensiveness
**Previous studies with linguistic minority groups** [16]	Views about harms caused by screening itself
	Forgetting to book screening appointments
	Lack of transparency on screening costs (some out-of-pocket)
	Wait times
	Making/getting to appointments
	Lack of awareness about retinopathy screening
	Language barriers
	Family and clinical support

The Canadian Ophthalmological Society recommends the implementation of tele-retinopathy screening programs to improve access to eye care in culturally, economically, or geographically isolated populations of individuals with diabetes [24, 25]. There are many examples of tele-retinopathy programs across Canada, and successful screening programs run in Newfoundland, Ontario, Quebec, Manitoba, Alberta, and British Columbia. The Toronto, Ontario, tele-retinopathy screening program, launched in September 2013, was developed to optimize retinopathy screening in Community Health Centers [26]. While promising, implementing a tele-retinopathy screening program in communities is unlikely to be sufficient to increase screening attendance for immigrants to Canada. Tele-retinopathy screening lends itself well to being supplemented with additional intervention strategies to address other attendance barriers/enablers [27].

The UK Medical Research Council’s guidance on the development and evaluation of complex interventions highlights the importance of thorough intervention development and feasibility assessment prior to evaluation and implementation [28]. Accordingly, in collaboration with health system and patient partners, we co-developed a culturally and linguistically tailored tele-retinopathy screening intervention with immigrants from China and African-Caribbean countries in Ottawa, Canada, enhanced to address barriers to screening attendance. The behavior change, theory-based intervention was co-developed over a period of 1 year by research partners, which included patient partners, health system partners, clinicians, and researchers via iterative co-design workshops [29]. The intervention was designed to target five prioritized barriers to attending retinopathy screening (language, knowledge of retinopathy, physician barriers regarding communication and support for screening, promotion about diabetic retinopathy screening, and fitting screening around other activities). The intervention includes behavior change techniques including instructions on how to perform a behavior, information about health consequences, social support (unspecified and practical), prompts/cues, adding objects to the environment, adding objects to the social environment, goal setting, action planning, problem solving, and restructuring the social environment. The intervention includes operationalized strategies and delivery channels that incorporate providing language support, pre-booking screening and sending reminders, social support via social media (i.e., WeChat and community champions), and providing resources such as posters, flyers, information sheets, and videos. Further details about the intervention content itself and its co-development are described elsewhere [29].

In our study, *acceptability* refers to the extent to which people living with diabetes from the two population groups who receive the intervention and health providers who deliver the intervention consider it to be appropriate [30, 31]. *Feasibility* in our study refers to the extent to which the tele-retinopathy screening intervention can be delivered for the given role and AACTT [32] (actor, action, context, time, target)-specified behavior of heath care providers involved in the implementation of the intervention and their experiences of barriers to and enablers of following the recommended procedure [33]. *Fidelity* in our study is reflected in the extent to which the intervention is implemented as planned [34] from the perspective of health care providers.

Our feasibility pilot study aims to:

1. Assess the feasibility, acceptability, and fidelity of an intervention to improve attendance to retinopathy screening delivered in a community health center for Mandarin and French-speaking individuals living with diabetes from China and African-Caribbean countries in Ottawa, Canada, over 6 months
2. Identify how many people from the two population groups attend the tele-retinopathy screening

## Methods

### Design and setting

We will work with our health system and patient partners to pilot the co-designed linguistically and culturally tailored tele-retinopathy screening intervention for Mandarin-speaking and French-speaking immigrants from China and African-Caribbean countries living with diabetes at a Community Health Centre in Ottawa, Canada. There is no existing retinopathy screening program at the community health center. The community health center houses general primary care practitioners and other health care providers, who provide services including diabetes education program and diabetes chiropody care for individuals living with diabetes. Services are provided in different languages and language interpretation services are available when required. We will use a triangulation mixed methods approach [35] to assess the feasibility, acceptability, and fidelity of the intervention. The Standard Protocol Items: Recommendations for Interventional Trials (SPIRIT) checklist [36] and the Consolidated Standards of Reporting Trials (CONSORT) extension for pilot and feasibility trials [37] were used as a guide for reporting this protocol. The SPIRIT checklist has been included as a supplementary file (Additional file 1).

### Participants and recruitment

#### Inclusion criteria

For this feasibility pilot, we will include adults with type 1 or type 2 diabetes who are Mandarin-speaking Chinese individuals or French-speaking African-Caribbean individuals with a screening referral from a physician or nurse practitioner and who self-report not having had a dilated eye examination in the last 12 months. We will exclude individuals who do not provide consent to participate in the research and refuse a dilated fundus examination.

#### Recruitment

##### Patient recruitment

Patients who attend the screening and meet our inclusion criteria will be invited by the screener to take part in the evaluation. Using a participant information sheet, patients will be informed about the evaluation, and we will obtain informed consent. We anticipate recruiting a total of 50–150 patient participants during the 6-month pilot assessment including individuals from both population groups. Since this is a feasibility pilot study, there is no power analysis for sample size determination. Part of our objectives is to quantify the attendance to the program itself, and our estimated sample size for patient recruitment aligns with sample sizes observed in other feasibility pilot studies (between 10 and 300 individuals) [38] and aligns with published recommendations regarding sample size for feasibility studies [39]. The anticipated sample size is also a function of the duration of the evaluation period (6 months) and available appointments for screening, assuming 1–2 patients attending screening each day.

##### Health care provider recruitment

We will invite each type of health care provider/administrator involved in the retinopathy screening program (screening site administrators, screening site managers, eye screening technicians, ophthalmologists interpreting images, and referrers involved in the delivery of the intervention) to participate in the evaluation. We aim to recruit at least 1 of each type of provider and thus anticipate recruiting about 5–10 health care providers/administrators. We will leverage our health system partners at the community center to reach out to prospective participants via email.

### Retinopathy screening intervention components and processes

The intervention includes components in the care pathway to address the prioritized barriers to attending retinopathy screening identified in our intervention co-development phase (i.e., language, knowledge of retinopathy, physician barriers regarding communication and support for screening, publicity about the screening, and fitting screening around other activities).

#### Tele-retinopathy screening attendance and procedures

##### Promotion to population groups

We will identify community health centers and family physicians in Ottawa that provide services to immigrants from China and African-Caribbean countries and inform them of the screening intervention. Based on our co-development work with health system and patient partners in the intervention development phase [29], potential patients will also be informed of the tele-retinopathy screening via promotion at walk-in clinics, community centers, places of worship, retail locations, and social settings often used by the two population groups. To address barriers around language, knowledge, and promotion about the screening attendance itself, we will use flyers, information sheets, posters, and videos (available in English, Mandarin, and French), which contain information about diabetic retinopathy, its importance, and instructions on how to access the screening intervention. Community champions (patient partners involved in co-developing the intervention and interested in facilitating the intervention promotion within the community groups) will use these materials to support individuals living with diabetes to attend the retinopathy screening intervention.

##### Referral and intake

Eligible patients will attend screening via referral from a physician, nursing practitioner, allied health workers (e.g., diabetes educators), or by self-referral. To address physician/health system barriers regarding communication and support for screening, when a patient self-refers for screening, the screening staff will use a standard template to facilitate obtaining physician referral, either from the patient’s primary care provider or from a physician/nursing practitioner affiliated with the screening intervention. The template will include a brief introduction to the intervention, a sign-off on the screening, and a sign-off to allow for the screening staff to book a specialist appointment, if necessary, post-screening. Obtaining a referral from patients’ primary care provider is preferred to ensure continuity of care.

##### Scheduling

The screening staff will schedule patient visits based on patient availability and the schedule and location of the camera. It is anticipated that the tele-retina screening camera may be moved between different sites to optimize patient access to services for the population groups. Also, the tele-retinopathy screening will be integrated into other diabetes programming such as foot care. This will enable fitting screening around other activities, which is a barrier our intervention aims to tackle.

##### Screening

An initial phone call will be made to the patient to obtain consent, collect demographic information, and schedule the appointment, followed by in-person screening. When patients arrive for screening, the screener trained in operating the imaging equipment will deliver elements of the intervention (e.g., reassurance of no harm caused to eyes due to screening) using the patient’s preferred language. In the event the screener does not speak the patient’s preferred language (i.e., French or Mandarin), translation support will be provided. The screener will take images of the retina using a portable retina camera (Topcon Maestro2 with color photos and OCT capabilities). Patients will be provided with linguistically and culturally tailored resources for evidence-based information about retinopathy (e.g., need for annual screening, risk factors, and information on what happens after screening). This will attend to language and knowledge of retinopathy barriers, as well as physician barriers regarding communication and support for screening.

##### Grading and access to images

Images will be uploaded to the Ontario Telemedicine Network (OTN)’s secure server, iVision tele-ophthalmology platform [40]. iVision is a robust tele-ophthalmology platform that complies with relevant privacy legislation and integrates ocular imaging, clinical workflows, and reporting into a single-source, cloud-based tele-retinal screening solution, which provides all the features necessary to manage the screening process. The images will then be securely forwarded or “uploaded” to an ophthalmologist/retina specialist for assessment, diagnosis, and/or treatment recommendations where needed. The retina specialists will grade the images, and a report generated in the OTN system will be accessible to the screening staff and forwarded to the referring health care provider. The report will include clear communication of the next steps and recommendations.

##### Patient follow-up after screening

**Fig. 1:**
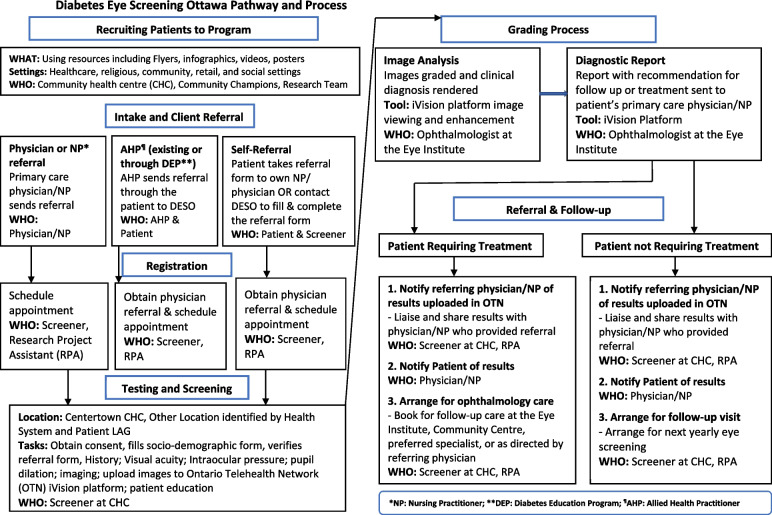
Tele-retinopathy screening pathway and process

The screening staff will notify the patient that the results of the screening have been shared with their primary care provider. Where a specialist appointment is needed, either the primary care provider or screening staff (depending on permissions provided on the referral form) will contact the patient to facilitate the next steps. Where further treatment is not required, the screening staff will schedule the next yearly tele-retinopathy screening, thus addressing physician barriers regarding communication and support for screening and enabling patient feedback. An outline of the tele-retinopathy screening process is presented in Fig. 1.

#### Screening staff training

All staff involved in screening patients will obtain training provided with the purchase of the imaging equipment regarding the operation of the tele-retina screening camera. Additionally, OTN will provide training about the use of the iVision platform and sending and accessing images securely. Furthermore, to ensure that screening personnel are adequately trained on the screening processes and the use of the equipment, they will receive training from our clinical research partners in Ottawa. The research team will train the screening staff in delivering other behavior change technique components of the intervention such as targeting knowledge barriers.

#### Pre-feasibility pilot

The screening intervention will first be tested in a pre-feasibility pilot study, whereby our health system and patient partners will test the intervention as designed. This will occur by running through the tele-retinopathy screening process with the intention to troubleshoot any challenges that arise and refine the screening intervention for the feasibility pilot.

### Feasibility pilot

#### Data collection

Consistent with the recommendations for feasibility pilot studies [39, 41, 42], we will focus data collection on the process, management, resources, and intervention. We will conduct a theory-based process evaluation alongside the delivery of the intervention to assess the feasibility and acceptability of attending the tele-retinopathy screening intervention.

#### Assessment of intervention acceptability by patients

While we have worked to co-develop a feasible intervention with patients from the communities that we seek to support in attending screening, there is a need to assess whether those actually engaging in the intervention itself find it acceptable. All eligible patients attending the screening will be invited to complete a post-intervention survey about their experience based on the Theoretical Framework of Acceptability (TFA) [30, 31] (Additional file 2). The survey will assess the acceptability of the intervention by including items related to each domain of the TFA: affective attitude (emotions related to intervention), burden (effort involved), ethicality (consistent with values), intervention coherence (clarity on how the intervention functions), opportunity costs (what they had to provide to take part), perceived effectiveness (whether the intervention is likely to work), and self-efficacy (confidence that they will get screened again). The response format of the survey will include both Likert scales and open-ended statements. To assess feasibility among patients, the survey will also explore patient-related costs as a result of attending the intervention such as travel and parking costs. Surveys will be available in English, Chinese, and French. Patient participants will complete the survey after screening via Microsoft Forms using an iPad device that will be available at the screening. A community health center volunteer will be present to assist patients in completing the survey, if needed. It will take about 15 min to complete the survey. Responses in Chinese or French will be translated into English prior to analysis. A mean score above the middle of the scale for each TFA measure, for each group will inform interpretation of the acceptability of the intervention from the perspective of patients.

#### Assessment of feasibility, acceptability, and fidelity of delivery by health care providers

We will assess the acceptability of the intervention, barriers and enablers experienced, and fidelity of delivery with health care providers involved in delivering the intervention. To evaluate feasibility, we will conduct interviews using a semi-structured interview topic guide (Additional file 3) informed by the Theoretical Domains Framework (TDF). Interviews will be used to assess the barriers and enablers to delivering the intervention for the given role and AACTT [32] (actor, action, context, time, target)-specified behavior of each health care provider involved in the implementation of the intervention (e.g., barriers to referring patients from a primary care physician perspective and barriers to delivering intervention from a screener perspective). We will assess feasibility based on the extent to which the barriers experienced were or could be addressed. The individuals who will execute the study protocol and complete the feasibility interviews at the community health center are dedicated staff who will only see study patients. On the other hand, ophthalmologists/retina specialists who will be interviewed are not dedicated to seeing only study patients and may need to adapt to using a new care workflow for patients who meet study enrollment criteria, while continuing to perform their other job duties.

The interview guide will also include a brief survey assessing the acceptability of the intervention informed by the TFA [30] and another survey assessing the fidelity of the intervention delivery informed by the National Institutes of Health (NIH) framework on assessing intervention fidelity [34] (Additional file 3). The response format of the surveys will include both Likert scales and open-ended statements. For acceptability, a median score of mid-point of the scale or above for each TFA measure will inform our interpretation of whether we think the intervention is acceptable from the perspective of the staff involved. We will assess whether the intervention as a whole was delivered with fidelity from the perspective of different delivery roles, based on a median score of mid-point of the scale or above for each indicator of fidelity reported on the following: provider training sufficiency, adherence to intervention protocol, and adaptations made. For questions related to adherence to intervention protocol and adaptations made, we will also prompt respondents to elaborate on any discrepancies and adaptations made. We will combine these self-reports with tracked attendance to training (number of attendees at each training event).

One-on-one interviews will be conducted virtually via Zoom at months 1 and 6 of the intervention pilot. The same health center staff and care delivery personnel will be interviewed at both months 1 and 6. Interviews will take approximately 1 h and will be audio recorded, transcribed with direct identifiers removed, and then analyzed.

### Intervention related data

In addition, during the feasibility pilot study, we will track referrals, including how many patients were referred to the intervention and time between the request for physician referral and referral receipt. We will also track the duration of screening, number of patients attending, dropout rates, time between screening and ophthalmologist interpretation assessment/diagnosis, and follow-up treatment recommended. Furthermore, an intake questionnaire administered to patients will collect information on age, insurance coverage, diabetes type, insulin use, age at diagnosis of diabetes, reported HbA1c, existing co-morbidities, last dilated eye examination, other ocular diseases, country of origin, preferred languages, and years since arriving in Canada. These data will allow us to estimate how many Mandarin-speaking and French-speaking individuals living with diabetes from China and African-Caribbean countries in Ottawa attend the tele-retinopathy screening intervention.

### Data analysis

For quantitative data, we will report means and standard deviations for parametric data, medians, and inter-quartile ranges for non-parametric data. Demographic characteristics (such as age, gender) will be summarized using frequency tables (*n* (%)) for categorical variables. The patient survey responses will be descriptively analyzed using IBM SPSS Statistics 25.0. We will run descriptive statistics to report means and standard deviations of responses to each TFA construct, and descriptively report categorical data (e.g., demographic characteristics) in terms of frequencies and percentages, for each group. Any open-ended responses will be analyzed qualitatively using directed content analysis based on TFA constructs [30, 31]. Descriptive analysis at months 1, 3, and 6 will inform feasible iterations of the intervention based on these data. If the mean scores on any TFA construct for either population group are below the mid-point of the scale at months 1 and 3, we will use the findings to suggest potential additional strategies and address concerns in the intervention, but we will continue running the program given that it is an entirely new screening program. If the mean scores on any TFA construct are below the mid-point of the scale at month 6 (final feasibility evaluation time point), we will take this to be an indicator that some aspect(s) of the intervention should be further iterated for improved acceptability. If that is the case, we will convene a patient and health system advisory group to decide on whether and how to continue the program beyond the 6-month feasibility pilot and decide on approaches for optimization of the program and its delivery.

The feasibility outcomes obtained from the interviews with health care providers will be analyzed using content analysis guided by the TDF [43] using NVivo. Data codes will be generated by labeling one to two lines of text with a descriptive label and then subsequently sorting these into the TDF domains. Data will be compared within and across codes to assess the similarities, differences, and interrelations and refined accordingly. Codes representing similar thematic topics will be grouped; these will be defined and documented in a codebook. Interview transcript analysis will involve the following four steps: (1) familiarization; (2) coding participant responses to specific domains, as defined by the TDF; (3) generating sub-themes within each domain; and (4) grouping themes across domains [33, 44]. To verify the emerging analysis, a second analyst will review a preliminary set of themes to assess how well the data are represented and the relevance of data within codes and to the associated TDF constructs. Where differences in interpretation arise, the two analysts will discuss until agreement is reached and amendments will be made to the coding and codebook as necessary. The health care provider survey responses from the interviews will be descriptively analyzed and scale scores calculated, and open-ended responses will be summarized based on the TFA constructs and NIH framework.

To document and analyze the costs related with the tele-retinopathy intervention, we will obtain the intervention and implementation costs [21] from the community health center. The intervention cost includes expenses required to purchase and maintain the intervention, salaries for staff, and physician fees. We will use these data along with downstream costs incurred (i.e., patient-related costs, such as travel and parking costs as well as time missed from work), to inform future economic evaluation of the intervention.

## Discussion

We will conduct a 6-month feasibility pilot of a behavior change, theory-based linguistically and culturally tailored tele-retinopathy screening intervention for Mandarin-speaking immigrants from China and for French-speaking immigrants from African-Caribbean countries living with diabetes delivered in a community health center in Ottawa, Canada. The intervention was co-developed with patient and health system partners to increase attendance to retinopathy screening and includes operationalized strategies and delivery channels. The findings from this feasibility pilot study will identify how many individuals living with diabetes from the target population groups attended the tele-retinopathy screening intervention, show how feasible and acceptable the intervention is, and demonstrate to what extent the intervention was delivered as planned. The study outcomes will foster an understanding of the experiences of patients in accessing and attending the screening and of health care providers in delivering the intervention, as well as enable the optimization of the community-based tele-retinopathy intervention.

By making transparent the experience of the staff and patients in terms of their perceptions of feasibility and acceptability, and through our assessment of fidelity, adaptation, and frequency of screening, our hope is that other jurisdictions considering developing a new tele-retinopathy screening program in general, and especially those tailored to language and culture, can look to the findings in this pilot for transferrable evidence. With our feasibility assessment among providers rooted in the TDF and assessed at two time points, we hope to understand any experienced barriers (and enablers) of those delivering the screening attendance intervention. Namely, what early barriers/enablers might have come up, whether any persist, and whether any adaptations were made to address any barriers. A further feasibility consideration among providers will be the planned cost analysis. With our feasibility assessment with patients, we are focusing specifically on investigating any patient-incurred costs, to help assess whether there may be future adaptations to the intervention for a planned trial that could mitigate these. With our acceptability assessment, we will have two measurement points of data with health providers on items assessing constructs from the theoretical framework of acceptability, providing us with an opportunity to descriptively assess variation on views about intervention coherence, perceived effectiveness, ethicality, self-efficacy, burden, opportunity costs, affective attitude, and general acceptability. The results will help to inform any additional refinement to the intervention components and staff support and training if any are flagged to have opportunities for greater acceptability. The same TFA data collected with patients over the 6 month pilot period will enable us to compare each of our population groups. With our fidelity assessment, we hope to learn not only what was delivered as planned but also what adaptations may have been put in place. This will help to decide whether future versions of the intervention should incorporate these adaptations to inform a wider-scale trial and any application of this program in other jurisdictions. Overall, we will apply this to understand the considerations for adding, removing, or formally modifying elements of the intervention for a future trial in collaboration with health system stakeholders and patients.

We anticipate that this study will improve access and increase attendance to diabetic retinopathy screening among Mandarin and French-speaking immigrants from China and African-Caribbean countries with diabetes, which will ultimately support a reduction of preventable blindness in Canada. The findings from this study will inform the implementation of tele-retinopathy screening programs with other ethnic minority groups, a future large-scale pragmatic randomized trial and economic evaluation of the linguistically and culturally tailored tele-retinopathy screening program.

### Supplementary Information

**Additional file 1. **SPIRIT checklist.

**Additional file 2. **Post screening survey for patients.

**Additional file 3. **Interview guide for healthcare providers.

## Data Availability

The datasets used and/or analyzed during the current study will be available from the corresponding author upon reasonable request.

## Abbreviations

TDF: Theoretical Domains Framework
TFA: Theoretical Framework of Acceptability
OCT: Optical coherence tomography
OTN: Ontario Telemedicine Network
