# Supplementary material for: Assessing the feasibility, acceptability, and fidelity of a tele-retinopathy-based intervention to encourage greater attendance to diabetic retinopathy screening in immigrants living with diabetes from China and African-Caribbean countries in Ottawa, Canada: a protocol
Source: Pilot Feasibility Stud. 2023 Sep 9;9:158. doi: 10.1186/s40814-023-01372-5 (PMC10492373; doi:10.1186/s40814-023-01372-5)
Supplement: Supplementary file 2 — Additional file 2. Post screening survey for patients. [file 40814_2023_1372_MOESM2_ESM.docx]

# **Additional file 2. Post screening survey for patients**

We would like to understand your screening experience so we can know if the program is suitable, aligns well with you, and is able to increase diabetic retinopathy screening for French speaking African-Caribbean persons/ Mandarin speaking Chinese people living with diabetes in Ottawa

- Please answer the questions based your experiences. There is no right or wrong answer
- Kindly provide extra feedback in the comment boxes
- All information provided will remain confidential
- It will take about 15 minutes to complete this survey

**Part 1: Acceptability Questionnaire**

| **Perceived effectiveness** | | |
| --- | --- | --- |
| 1 | The program has improved access for me to attend diabetes eye screening | Strongly disagree (1), disagree (2), Neither agree nor disagree (3), Agree (4), Strongly agree (5) |
|  | Comments |  |
| **Intervention coherence** | | |
| 2 | It is clear to me how the diabetes eye screening program will help prevent eye problems due to diabetes | Strongly disagree (1), disagree (2), Neither agree nor disagree (3), Agree (4), Strongly agree (5) |
|  | Please tell us more about your views |  |
| **Ethicality** | | |
| 3 | How fair is the eye screening program for African-Caribbean persons/ Mandarin speaking Chinese people living with diabetes? | Very unfair (1), Unfair (2), No Opinion (3), Fair (4), Very fair (5) |
|  | Please explain |  |
| **Self-Efficacy** | | |
| 4 | How confident do you feel about attending the eye screening program again? | Very unconfident (1), Unconfident (2), No Opinion (3), Confident (4), Very confident (5) |
| **Burden** | | |
| 5 | How much effort did it take to attend the eye screening? | No effort at all (1), A little effort (2), No opinion (3), A lot of effort (4), Huge effort (5) |
| **Opportunity Costs** | | |
| 6 | Attending the eye screening interfered with my other priorities | Strongly disagree (1), disagree (2), Neither agree nor disagree (3), Agree (4), Strongly agree (5) |
|  | Comments |  |
| **Affective Attitude** | | |
| 7 | How comfortable did you feel about attending the diabetes eye screening program? | Very uncomfortable (1), Uncomfortable (2), No Opinion (3), Comfortable (4), Very comfortable (5) |
| **General Acceptability** | | |
| 8 | How acceptable was the diabetes eye screening program to you? | Completely unacceptable (1), Unacceptable (2), No Opinion (3), Acceptable (4), Completely acceptable (5) |
| 9 | Overall, how was your experience with the screening? | Very poor experience (1), Poor experience (2), No Opinion (3), Good experience (4), Very good experience (5) |
| 10 | Is there anything else about the diabetes eye screening program that you would like to mention? (Other comments) |  |

**Part 2: Patient Cost Questionnaire**

Thinking about the **Diabetes Eye Screening**

1. How much of **your own money** did you pay for the visit and diagnostic tests?

 None OR enter amount: $_________

1. How long did it take you to travel to the community centre?

________ hours ________minutes

1. Approximately what distance did you have to travel to get to the community centre (one-way)?

_______________miles

1. Travel (s) for the eye screening
   1. How did you travel/get to the community centre? Please tick the **main forms of transport**

| Walking | □ |
| --- | --- |
| Private car | □ |
| Public transport | □ |
| Taxi | □ |
| Other | □ Please specify……………………… |

- 1. If you traveled by **a private car**, were you given a lift by someone else?

□ Yes □ No

- 1. If you traveled by **a private car**, how much was paid in car park fees?

_______________$

- 1. If you traveled by **public transport (bus or train)**, what was the cost of **the one-way fare**? If you were given a return fare, halve it. Put zero if you did not travel by public transport at all or you did not pay a fare

_______________$

- 1. If you traveled by **taxi,** what was the cost of the **(one-way) fare**? Put zero if you did not travel by taxi at all, or you did not pay a fare.

_______________$

1. Did anyone **accompany you to the community centre and wait for you** while you received eye screening?

□ Yes □ No

If Yes, did they take time off their paid work? □ Yes □ No

For how many hours?_______________ hours

1. If you have other dependants, did you **pay** someone to look after them?

□ Yes □ No □ Not applicable

If Yes, how much did it cost? _______________$

1. How long did you spend waiting at the community centre before your appointment?

________ hours ________minutes

**Part 3: Productivity Cost Questionnaire**

The following questions ask about the effect of the **Diabetes Eye Screening** on your ability to work and perform regular activities. Please fill in the blanks or circle a number, as indicated.

1. What would have been your **main activity** if you had not attended the eye screening?

| Paid employment | □ |
| --- | --- |
| Looking after relatives | □ |
| Leisure activities | □ |
| Housework | □ |
| Studying at college/university | □ |
| Other | □ Please specify…………………………… |

If you are in paid employment, please answer question 9, if not go to question 12.

1. During the past seven days, how many hours did you work?

______hours (If "0", skip to question 12)

1. What arrangements did you make to take time off work? (Please tick one box)

| Paid absence from work | □ |
| --- | --- |
| Unpaid absence from work | □ |
| Will make the time up | □ |
| Came to the eye screening outside work time | □ |
| Took holiday | □ |
| Other arrangements | □ Please specify………………… |

1. How many hours did you miss from work because of the **Diabetes Eye Screening**? Include hours you missed, times you went in late, left early, etc., because of the screening.

_____ hours

1. If you have any comments about your costs for attending the screening, please write them below

____________________________________________________________________________

**BACKGROUND INFO**

**Thank you for sharing your views and experience. To conclude, we would like to collect a few background details so that we can make sure different views are captured in our study and to better describe who is in our study in a general sense.**

1) What is your age?

2) How would you describe your gender identity?

□ Male □Trans (female to male) □ Other (please specify):______

□ Female □Trans (male to female) □ Prefer not to answer

□ Intersex □ Two-Spirit

3) What language do you feel most comfortable in when speaking with your health care providers?

□ Arabic □ Farsi □ Portuguese □ Urdu

□ Bengali □ French □ Punjabi □ Vietnamese

□ Chinese (Cantonese) □ Greek □ Spanish □ Other (specify):

□ Chinese (Mandarin) □ Hindi □ Tagalog □ Prefer not to answer

□ English □ Korean □ Tamil

4) Were your parents born in Canada? □ Yes □ No □ Prefer not to answer

If NO, in what country were they born?: ______________

5) What is your source of income? (check all that apply)

| □Full time employment | **Government Assistance:** | □No income |
| --- | --- | --- |
| □Part time employment | □Old Age Security (OAS) | □Other (please specify): _______ |
| □Student loan | □Ontario Works (OW) | □Do not know |
| □Canadian Pension Plan (CPP) | □ Employment Insurance (EI) | □Prefer not to answer |
| □Retirement income | □Ontario Disability Support Program (ODSP) |  |

6) Are you part of diabetes education program? □ Yes □ No

If Yes, please specify: ___________________

7) How did you hear about the screening?

□ Primary Care Provider □ Diabetes Education program □ SCREEN Program

□ Centertown Community Health Centre □ Other Community Centre (please specify): ________

□ WeChat □ Website (please specify): ___________ □ Other (please specify): ___________

**Thank you for sharing your experience and your participation!**
