# Supplementary material for: Assessing the feasibility, acceptability, and fidelity of a tele-retinopathy-based intervention to encourage greater attendance to diabetic retinopathy screening in immigrants living with diabetes from China and African-Caribbean countries in Ottawa, Canada: a protocol
Source: Pilot Feasibility Stud. 2023 Sep 9;9:158. doi: 10.1186/s40814-023-01372-5 (PMC10492373; doi:10.1186/s40814-023-01372-5)
Supplement: Supplementary file 3 — Additional file 3. Interview guide for healthcare providers. [file 40814_2023_1372_MOESM3_ESM.docx]

**Additional file 3. Interview guide for healthcare providers**

We want to hear from health care providers involved in the delivery of the diabetes eye screening program. We want to hear your thoughts on the program’s feasibility, acceptability, and the extent to which the program is/was implemented as planned, which will help to evaluate the program. If there were any issues experienced, we really want to hear about them so we can improve the program.

The interview will take about an hour. Feel free to stop the conversation at any time, take a break, or not answer questions you’re not comfortable answering. It is completely up to you whether you want to participate in this study. Do you have any questions before we start?

To begin,

What is your role in the Diabetes Eye Screening Program?

**1. I would like** **to hear a little bit about what you know about the screening program for individuals from China and African/Caribbean countries living with diabetes. [KNOWLEDGE]**

1. How would you describe the diabetes eye screening program and how it works?
2. What do you think is the goal of the screening program?
3. What action(s) was required of you to participate in the screening program? (e.g. obtain training, provide education)
4. What are the likely ways persons living with diabetes can attend the screening?
5. What strategies do you currently use to make individuals from China and African/Caribbean countries living with diabetes aware of the program?

**2. Can you think of anything in your environment that might affect how you deliver the screening program? [ENVIRONMENTAL CONTEXT AND RESOURCES]**

1. To what extent does your working environment have sufficient resources needed to allow you to perform the duties associated with the screening program?
2. How, if at all, does your working environment support you in performing your duties?
3. What processes in the community centre prevent or enable you to deliver the screening program?
4. What resources do you think you will need to enable you to perform your duties?

**3. Have you delivered diabetes eye screening previously? Were you successful? Why or why not? [SKILLS]**

1. Can you think of skills or techniques you think might help you to deliver the screening program?

**4. How do your different roles (i.e., in your social and work life) or responsibilities affect how you deliver the screening [SOCIAL, PROFESSIONAL ROLE AND IDENTITY]**

1. What are the tasks or responsibilities you have that might affect how you deliver screening?
2. To what extent do you consider delivering the screening as part of your role?
3. What is unique about you that you think might influence how you deliver the screening program?

**5. How confident are you that you can deliver the screening program? What increases your confidence? What would reduce your confidence? [BELIEFS ABOUT CAPABILITIES]**

1. Are there any steps in the screening process that you feel more or less comfortable doing?
2. How much control do you think you have over delivering the program?

**6. What outcomes or effects have you noticed with the implementation of the screening program? Positive? Negative? [BELIEFS ABOUT CONSEQUENCES]**

1. For example, did the screening have any effect on the (self, patients, colleagues, the organization)?

**7. How confident are you about the diabetes eye program being a success? What is your role in this? [OPTIMISM]**

**8. What sort of experiences would you find rewarding and encourage you to deliver the screening program? [REINFORCEMENT]**

a. Have you found anything rewarding/discouraging whilst supporting the program?

**9. How is the delivery of the diabetes eye screening program prioritized among your responsibilities? Do you want to deliver the screening? How much effort would you want to invest? [INTENTION]**

a. To what extent do you intend to continue delivering retinopathy screening in practice?

**10. What are other goals/priorities that might get in the way of delivering the screening program? [GOALS]**

**11. How easy or difficult is it to remember the steps and processes involved in performing the diabetes eye screening? [MEMORY, ATTENTION AND DECISION PROCESSES]**

a. Were there any situations in which you decided not to deliver some component of the program?

**12. How, if at all, do you think that other people can affect how you deliver the screening program? If at all, who might affect you in delivering the program? [SOCIAL INFLUENCES]**

a. Are there conflicting beliefs among your colleagues about the diabetes eye screening program? If so, what are they?

**13. How do you feel about the diabetes eye screening program? To what extent do you feel that your emotional state affects your ability to deliver the screening program? [EMOTION]**

a. Did you enjoy taking part in the program? If yes, what did you enjoy about it? If no, why not?

**14. What do you think might help you to deliver the screening program? [BEHAVIOURAL REGULATION]**

a. Do you receive feedback on your support of the screening program?

b. What other actions do you do to support the screening?

15. **Given everything you raised as barriers, were there any that you weren’t able to address or any that you think could not be addressed that may impact on how feasible it was to do this?**

**TFA**

To further understand the acceptability of the diabetes eye screening program, please provide your responses to the following statements:

| **Intervention coherence** | | |
| --- | --- | --- |
| 1 | It is clear to me how the diabetes eye screening program will help improve diabetes care | Strongly disagree (1), disagree (2), Neither agree nor disagree (3), Agree (4), Strongly agree (5) |
|  | Tell us more about your views |  |
| **Perceived effectiveness** | | |
| 2 | The screening has improved access to diabetic eye screening for French speaking African-Caribbean and Chinese immigrants to Canada | Strongly disagree (1), disagree (2), Neither agree nor disagree (3), Agree (4), Strongly agree (5) |
|  | Comments |  |
| **Ethicality** | | |
| 3 | There are moral or ethical consequences in delivering the screening program | Strongly disagree (1), disagree (2), Neither agree nor disagree (3), Agree (4), Strongly agree (5) |
|  | Please explain |  |
| **Self-Efficacy** | | |
| 4 | How confident do/did you feel about delivering the screening program within your role? | Very unconfident (1), Unconfident (2), No Opinion (3), Confident (4), Very confident (5) |
| **Burden** | | |
| 5 | How much effort does/did it take to deliver the screening? | No effort at all (1), A little effort (2), No Opinion (3), A lot of effort (4), Huge effort (5) |
| **Opportunity Costs** | | |
| 6 | Delivering the retinopathy screening interfered/would interfere with my other priorities | Strongly disagree (1), disagree (2), Neither agree nor disagree (3), Agree (4), Strongly agree (5) |
|  | If so, in what way? |  |
| **Affective Attitude** | | |
| 7 | How comfortable do/did you feel delivering the diabetes eye screening program? | Very uncomfortable (1), Uncomfortable (2), No Opinion (3), Comfortable (4), Very comfortable (5) |
| **General Acceptability** | | |
| 8 | How acceptable is/was the diabetes eye screening program to you? | Completely unacceptable (1), Unacceptable (2), No Opinion (3), Acceptable (4), Completely acceptable (5) |

**FIDELITY**

We would like to explore the extent to which the program is/was implemented as planned. Please provide your responses to the following statements:

| **Training Providers** | | |
| --- | --- | --- |
| 1 | The training provided enabled me carry out my role in the screening program | Strongly disagree (1), disagree (2), Neither agree nor disagree (3), Agree (4), Strongly agree (5) |
| **Ensure adherence to intervention protocol and Reduce differences within intervention** | | |
| 2 | There were discrepancies between what was intended to be carried out in the diabetes eye screening program and what was done (referral/patient intake, screening, grading, reporting, and follow-up) | Strongly disagree (1), disagree (2), Neither agree nor disagree (3), Agree (4), Strongly agree (5) |
|  | If so, what were the discrepancies? |  |
| **Control for provider differences** | | |
| 3 | Within my role, each essential aspect of the screening is delivered as intended | Strongly disagree (1), disagree (2), Neither agree nor disagree (3), Agree (4), Strongly agree (5) |
| 4 | I made adaptations to the screening process | Strongly disagree (1), disagree (2), Neither agree nor disagree (3), Agree (4), Strongly agree (5) |
|  | If so, what were the adaptations? And why were adaptations made? |  |

Do you have any other thoughts or comments that would assist in the DRS program evaluation?

**BACKGROUND INFO**

**Thank you for sharing your views and experience. To end our conversation today, if it is okay with you, I would like to collect a few background details so that we can make sure different views are captured in our study and to better describe who is in our study in a general sense.**

1. What is your age?
2. How would you describe your gender identity?

□ Male                   □Trans (female to male)             □ Other (please specify):______

□ Female               □Trans (male to female)              □ Prefer not to answer

□ Intersex              □ Two-Spirit

1. Years of experience in your field?
2. Are there any communities that you identify with that you would like to mention, such as ethnic or racial communities, or any others?
3. What is your first language?

□ Arabic                          □ Farsi                □ Portuguese              □ Urdu

□ Bengali                        □ French             □ Punjabi                    □ Vietnamese

□ Chinese (Cantonese)   □ Greek              □ Spanish                    □ Other (specify):

□ Chinese (Mandarin)    □ Hindi               □ Tagalog                   □ Prefer not to answer

□ English                       □ Korean             □ Tamil

**Thank you very much for sharing your thoughts with me, they will be extremely helpful for us to consider as we continue our project to help improve health and well- being and increase diabetes eye screening for Chinese and Francophone African-Caribbean persons with diabetes in Ottawa.**
